# *CDCA8* and *TROAP* as Prognostic Biomarkers of Postoperative Metastatic Progression in Clear Cell Renal Cell Carcinoma

**DOI:** 10.3390/cancers17182975

**Published:** 2025-09-11

**Authors:** Mingyu Kim, Geehyun Song, Jaeyoung Joung, Hokyung Seo, Hyungho Lee, Jinsoo Chung

**Affiliations:** 1 Center for Urologic Cancer, National Cancer Center, 323, Ilsan-Ro, Ilsandong-Gu, Gyeonggi-Do, Goyang-Si 10408, Republic of Korea; kmk63819@ncc.re.kr (M.K.);; 2 Department of Urology, Urological Cancer Center, National Cancer Center, Goyang-Si 10408, Republic of Korea

**Keywords:** ccRCC, biomarker, *CDCA8*, *TROAP*

## Abstract

Objectives: Clear cell renal cell carcinoma (ccRCC) may later metastasize despite curative surgery. This study asked whether transcriptomic alterations detectable at nephrectomy are associated with subsequent metastatic progression, and whether such signals retain prognostic relevance in overt metastatic disease. Methods: Bulk RNA sequencing was performed in 30 ccRCC patients without metastasis at surgery; 4 developed distant metastasis during follow-up. Differential expression, enrichment, and network analyses identified hub genes, which were screened by ROC analysis with bootstrap optimism correction. External validation used TCGA-KIRC focusing on patients metastatic at baseline (M1) to evaluate overall and disease-specific survival with multivariable Cox models (per-SD expression, adjusted for age, sex, and stage); Kaplan–Meier curves were shown for visualization only. Results: Fifty-nine DEGs distinguished patients who later metastasized from those who remained metastasis-free, with enrichment in mitotic and chromosomal-segregation pathways. Five hub genes (*BASP1*, *CDCA8*, *KIF2C*, *LMNB1*, *TROAP*) showed high discrimination in the discovery set (optimism-corrected AUC ~0.92–0.93). In TCGA-M1, *CDCA8*, and *TROAP* were consistently associated with worse survival and remained significant in multivariable models. Conclusions: Dysregulation of mitotic control may underlie latent metastatic competence in ccRCC. *CDCA8* and *TROAP* emerge as candidate prognostic biomarkers, linking postoperative metastatic progression in an initially M0 cohort with survival in metastatic disease. These hypothesis-generating findings warrant validation in larger, prospective cohorts.

## Simple Summary

Kidney cancer known as clear cell renal cell carcinoma (ccRCC) often appears localized at the time of surgery, but some patients later develop metastasis. This study asked whether patterns of gene activity in the original tumor can reveal which patients are at higher risk. We analyzed RNA from tumors of 30 patients who had no metastasis at surgery and followed them over time; 4 later developed distant spread. By comparing gene expression and groups of interacting genes, we found a set of cell-division-related genes that differed between patients who did and did not later metastasize. Two genes, *CDCA8* and *TROAP*, consistently marked higher risk and were linked to worse survival in an independent dataset. These findings suggest that early changes in cell-cycle control may underlie later spread, and that *CDCA8* and *TROAP* could help guide postoperative risk assessment and follow-up planning.

## 1. Introduction

ccRCC is the most common histological subtype of kidney cancer, accounting for approximately 75–80% of renal malignancies [1,2,3]. Despite advances in surgery and systemic therapy, ccRCC remains a clinically heterogeneous disease with variable outcomes [4,5,6,7]. For patients with localized tumors, nephrectomy offers curative potential [8,9], yet a substantial proportion subsequently experience distant relapse, often years after surgery despite an initially metastasis-free status [10,11,12]. This latent progression highlights the biological complexity of ccRCC and underscores the need for more precise biomarkers to identify patients with aggressive disease biology who are at higher risk of poor outcomes [13,14,15]. Conventional prognostic systems such as Tumor Node Metastasis (TNM) stage and Fuhrman grade provide limited capacity to detect micrometastatic potential, as patients with similar clinical features often show markedly different trajectories [16,17,18,19,20,21]. High-throughput profiling studies have begun to uncover molecular subtypes and immune- or cell-cycle-related signatures associated with ccRCC progression [22,23,24]. However, most investigations compared primary and metastatic tumors, which may conflate intrinsic metastatic programs with treatment-related or disease-burden effects [25,26]. A more clinically relevant approach is to analyze transcriptomic alterations in tumors that are localized at surgery but later metastasize, thereby revealing molecular features associated with aggressive disease [27,28]. Yet, such longitudinally annotated cohorts remain scarce due to the difficulty of assembling adequate follow-up data. In this study, we profiled tumors from 30 ccRCC patients who had no evidence of metastasis at nephrectomy, among whom 4 subsequently developed distant recurrence. Although the small number of metastatic events limits the capacity for robust prediction, exploratory analysis revealed transcriptional programs enriched in mitotic checkpoint and chromosomal stability pathways. Among the hub genes, *CDCA8* and *TROAP* emerged as consistently upregulated, consistent with their known roles in chromosome segregation and cell-cycle regulation. These genes have also been linked to tumor aggressiveness in other cancers [29,30], but their prognostic relevance in ccRCC has not been systematically evaluated. To further assess their clinical significance, we validated their association with survival in the TCGA cohort, where high expression correlated with poorer outcomes in metastatic patients. Our findings highlight *CDCA8* and *TROAP* as potential prognostic biomarkers that reflect aggressive disease biology and may assist in postoperative risk stratification for ccRCC.

## 2. Materials and Methods

### 2.1. Patient Cohort and Clinical Information

This study included 30 patients diagnosed with clear cell renal cell carcinoma (ccRCC) who underwent radical or partial nephrectomy at the National Cancer Center. All patients had no radiological or pathological evidence of distant metastasis (M0) at the time of surgery. During longitudinal follow-up, 4 patients subsequently developed distant metastasis, while the remaining 26 remained metastasis-free. Detailed clinicopathological features are summarized in Table S1 (in Supplementary Materials). Given the small number of metastatic events, analyses in this cohort were regarded as exploratory and primarily intended to identify candidate biomarkers for external validation. Written informed consent was obtained from all participants, and the study protocol was approved by the National Cancer Center Institutional Review Board (IRB No. NCC2021–0147).

### 2.2. Transcriptomic Data Generation and Differential Expression Analysis

Total RNA was extracted from fresh-frozen tumor tissue samples using the RNeasy Mini Kit (Qiagen, Hilden, Germany). RNA quality and integrity were assessed with the TapeStation RNA ScreenTape (Agilent Technologies, Santa Clara, CA, USA), and only samples with RNA integrity number (RIN) > 7.0 were used. RNA libraries were constructed using the Illumina TruSeq Stranded mRNA Library Prep Kit (Illumina, San Diego, CA, USA) and paired-end sequencing (2 × 100 bp) was performed on the Illumina NovaSeq 6000 platform (Macrogen Inc., Seoul, Republic of Korea). Raw FASTQ reads were aligned to the human genome reference (GRCh38) using STAR aligner, and transcript quantification was performed with RSEM to generate TPM and expected count matrices. Downstream analyses were conducted in R (v4.4.3) using DESeq2 [31]. Genes with row sums < 10 were filtered. Differential expression analysis between patients who later developed metastasis and those who remained metastasis-free was performed using DESeq2’s negative binomial model. DEGs were defined as those with adjusted *p*-values < 0.05 and |log2FC| > 1. These comparisons were conducted in an exploratory manner to highlight transcriptional alterations potentially associated with aggressive disease biology.

### 2.3. Assessment of Technical Variation

To examine whether technical artifacts could have contributed to the observed clustering patterns, we evaluated sequencing quality control (QC) metrics, including total read count and GC content, for each sample. Principal component analysis (PCA) was first performed using TMM-normalized expression data. The resulting principal components were then annotated with QC metrics, and potential associations were assessed both visually and statistically. Specifically, PCA plots were colored by sequencing depth and GC content, and Spearman correlation tests were conducted between the first five principal components (PC1–PC5) and QC variables. All statistical analyses were conducted in R (v4.4.3).

### 2.4. Functional Network Analysis and Biomarker Evaluation

Functional enrichment of DEGs was performed using the clusterProfiler package (v4.8.1), including Gene Ontology (GO) terms across all three domains—biological process (BP), cellular component (CC), and molecular function (MF)—as well as MSigDB Hallmark gene sets. Genes without interaction partners or lacking enrichment annotations were excluded [31]. Hub genes were defined based on network connectivity and functional involvement in mitotic regulation or chromosomal segregation. Network construction and visualization were performed using Cytoscape (v3.10.0) [32], and enrichment plots were generated using enrichplot. To prioritize candidate biomarkers, ROC curve analysis was applied to all 22 hub genes using the pROC package (v1.18.5). Genes with 95% confidence intervals for AUC between 0.80 and 0.95 were selected. Expression differences between patients with and without subsequent metastasis were further assessed using the Wilcoxon rank-sum test. These analyses were considered exploratory and interpreted as hypothesis-generating rather than definitive predictive models.

### 2.5. External Validation and Prognostic Modeling

The TCGA-KIRC cohort was used to externally evaluate the prognostic relevance of the candidate genes [33]. Because TCGA does not provide uniformly curated information on the timing of postoperative metastasis or recurrence, the validation was designed to assess survival prognosis rather than recurrence risk. Patients annotated as metastatic at baseline (M1) were evaluated for overall survival (OS) and disease-specific survival (DSS). Gene expression values were standardized (z-score per gene), and multivariable Cox proportional-hazards models were fitted with expression as a continuous variable (per SD increase). Models were adjusted for age, sex, and AJCC stage (III–IV vs. I–II). Proportional hazards assumptions were assessed using Schoenfeld residuals, and hazard ratios (HRs) with 95% confidence intervals and two-sided *p*-values were reported. As a complementary analysis, multivariable Cox models were fitted in the full TCGA cohort with the same covariates plus an indicator for baseline metastasis status (M1 vs. M0) to examine whether gene–outcome associations were independent of baseline metastasis. This analysis also treated gene expression as a continuous (per-SD) variable. TCGA gene expression data (RSEM-normalized; log2-transformed where applicable) were standardized before modeling. Kaplan–Meier curves were generated for visualization only, while statistical inference was based on the continuous Cox models. Multiple testing correction was applied using the Benjamini–Hochberg procedure when appropriate. All analyses were performed in R version 4.4.3 (R Foundation for Statistical Computing, Vienna, Austria) using the survival, survminer, and related packages [34].

### 2.6. Statistical Analysis

All statistical analyses were performed using R (v4.4.3) and GraphPad Prism 10.3.1. Comparison between two groups were made using the Wilcoxon rank-sum test or unpaired two-tailed *t*-test, depending on data distribution. For multiple comparisons, *p*-values were adjusted using the Benjamini–Hochberg method. ROC analysis was conducted with the pROC package. To mitigate potential overfitting in the small discovery cohort, bootstrap resampling (1000 iterations) was applied to obtain optimism-corrected estimates of AUC and corresponding confidence intervals. A two-sided *p*-value < 0.05 was considered statistically significant. Figures and plots were generated using ggplot2, ComplexHeatmap, survminer, and enrichplot packages.

## 3. Results

### 3.1. Transcriptomic Landscape Distinguishes Future Metastatic Progression

**Figure 1:**
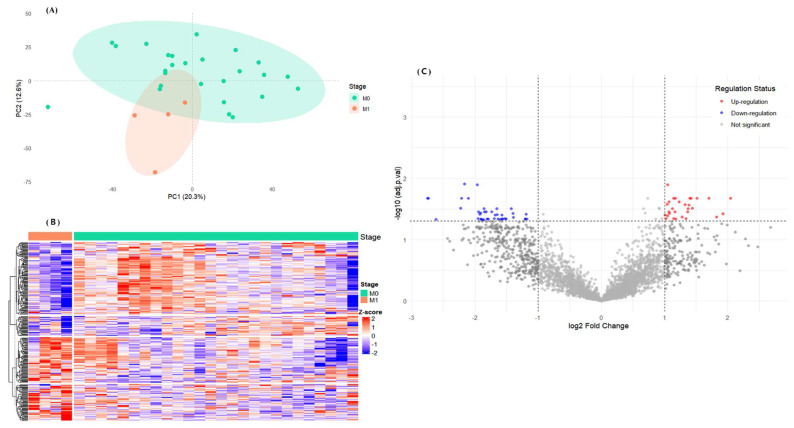
Transcriptomic landscape associated with postoperative metastatic progression in ccRCC. (**A**) PCA of bulk RNA-seq data shows partial separation between M0 (non-metastatic) and M1 (metastatic) patient groups, indicating underlying transcriptomic differences at the time of surgery. (**B**) Unsupervised hierarchical clustering based on the top DEGs reveal subgroup-specific expression patterns, supporting biological divergence between groups. (**C**) Volcano plot depicting DEGs between M1 and M0 groups (adjusted *p* < 0.05, |log_2_FC| > 1). Red and blue dots indicate significantly upregulated and downregulated genes in the M1 group, respectively.

To explore transcriptomic features associated with postoperative outcomes, RNA sequencing was performed on tumor tissues from 30 ccRCC patients who had no radiological or pathological evidence of distant metastasis (M0) at the time of nephrectomy. During longitudinal follow-up (median 23 months, range 11–33 months), 4 patients (13.3%) subsequently developed distant metastasis, while 26 remained metastasis-free (Table S1 in Supplementary Materials). This design enabled exploratory comparison of transcriptional alterations between patients who later experienced recurrence and those who remained disease-free, independent of overt tumor burden at surgery. Principal component analysis (PCA) of the whole-transcriptome profiles revealed a partial but visible separation between patients with and without subsequent metastasis, suggesting baseline molecular heterogeneity (Figure 1A). Unsupervised hierarchical clustering of the top-ranked differentially expressed genes (DEGs) further demonstrated consistent expression patterns within each subgroup (Figure 1B). Differential expression analysis using DESeq2 identified 59 DEGs between the two groups (adjusted *p* < 0.05, |log2FC| > 1), including 27 upregulated and 32 downregulated genes in patients who later developed metastasis (Figure 1C). These exploratory findings suggest that molecular programs related to mitotic regulation and chromosomal stability may already be detectable at the time of surgery in patients who subsequently demonstrate poorer outcomes. While limited by the small number of metastatic events, this discovery cohort provided candidate genes for external validation in larger datasets. To address potential concerns of hidden confounders or batch effects, the PCA was annotated with sequencing quality control (QC) variables (total read count and GC%). No clustering was observed according to these technical metrics (Figure S1, in Supplementary Materials). Correlation analyses between PC1–PC5 and QC variables identified modest but statistically significant associations for PC2 with GC% (ρ = –0.495, q_FDR = 0.027) and PC4 with read count (ρ = –0.516, q_FDR = 0.027; Table S2, in Supplementary Materials). These results indicate that QC variation explains only a limited component of variance and is unlikely to account for the primary separation observed between the clinical subgroups.

### 3.2. Functional Enrichment and Network Analysis of Metastasis-Associated Genes

**Figure 2:**
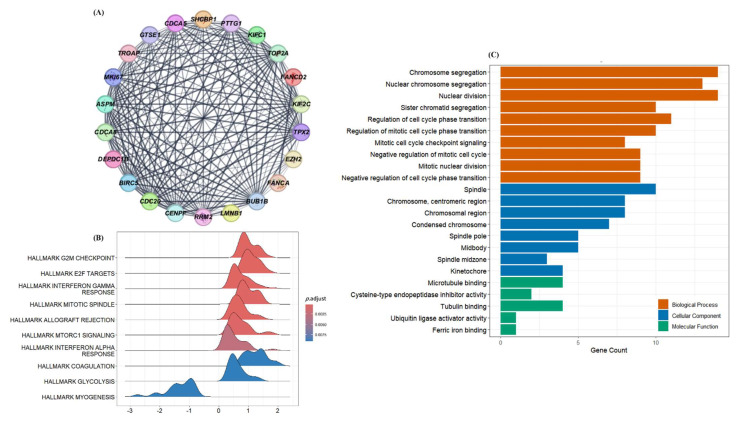
Functional enrichment and network-based identification of metastasis-associated hub genes. (**A**) Refined PPI network constructed from 59 DEGs using STRING and Cytoscape. A subset of 22 hub genes was selected based on combined criteria of network connectivity, cluster density, and functional annotation with mitotic regulation or chromosomal segregation pathways. (**B**) Hallmark pathway enrichment analysis of hub genes shows significant activation of cell-cycle-related processes, including G2/M checkpoint, E2F targets, and mitotic spindle assembly. (**C**) GO enrichment analysis visualizing biological processes enriched among hub genes. Key terms include chromosome segregation, spindle organization, and mitotic checkpoint signaling.

To gain mechanistic insights into the biological relevance of the DEGs, we performed a protein–protein interaction (PPI) network analysis using the STRING database. The 59 DEGs identified in Figure 1 were mapped onto the STRING network (confidence score > 0.4), which revealed extensive interconnections among a subset of genes involved in cell-cycle regulation, mitotic control, and chromosomal organization (Figure S2, in Supplementary Materials). To identify candidate drivers, we applied an integrative filtering strategy that combined network features with pathway-level evidence. Genes were not prioritized solely by topological metrics (e.g., degree centrality); instead, we retained those that showed (i) strong connectivity within dense interaction clusters, (ii) functional annotation with mitotic cell cycle or chromosomal segregation processes in enrichment databases such as Gene Ontology (GO) and Hallmark, and (iii) consistent differential expression between patients who did and did not develop metastasis. Genes with limited network integration and no pathway-level annotation were excluded from downstream analyses. This approach yielded 22 hub genes, including *CDCA8*, *TROAP*, *KIF2C*, and *LMNB1*, as visualized in the refined network (Figure 2A). Functional characterization of these genes confirmed their association with mitotic regulation: Hallmark analysis highlighted activation of E2F targets, G2M checkpoint, and mitotic spindle pathways (Figure 2B), while GO terms were enriched for chromosome segregation, mitotic checkpoint signaling, and kinetochore organization (Figure 2C). Collectively, these results suggest that dysregulation of mitotic control and chromosomal stability represents a recurrent feature of aggressive disease biology in ccRCC.

### 3.3. Selection of Robust Gene-Level Biomarkers Based on ROC Performance

**Figure 3:**
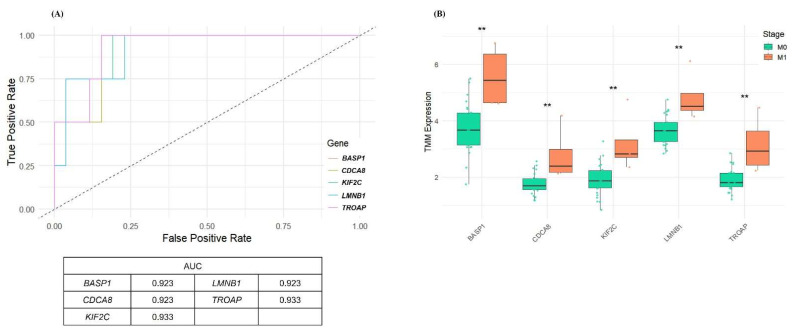
Diagnostic performance of selected hub genes in distinguishing postoperative metastasis. (**A**) ROC curves for five hub genes *BASP1*, *CDCA8*, *KIF2C*, *LMNB1*, and *TROAP* based on expression in M0 vs. M1 patients. All genes demonstrated high discriminative power with AUC values ranging from 0.923 to 0.933. (**B**) Boxplots comparing gene expression levels between M0 (non-metastatic) and M1 (metastatic) groups. All five genes showed significantly elevated expression in the M1 group (Wilcoxon rank-sum test, ** *p* < 0.01 indicates statistical significance).

To identify clinically meaningful biomarkers for postoperative outcomes, we evaluated the performance of all 22 hub genes using ROC curve analysis. Genes were selected if their 95% confidence intervals (CIs) for the area under the curve (AUC) were entirely within the range of 0.80 to 0.95, ensuring both discriminative ability and statistical reliability. To reduce the risk of overfitting in this small cohort, optimism-corrected AUC estimates were obtained using bootstrap resampling. This filtering identified five genes—*BASP1*, *CDCA8*, *KIF2C*, *LMNB1*, and *TROAP*—that consistently showed high classification performance, with optimism-corrected AUCs ranging from 0.92 to 0.93 (Figure 3A). Expression comparison further confirmed that all five genes were significantly upregulated in patients who later developed metastasis (*p* < 0.01, Wilcoxon test; Figure 3B). Among them, *CDCA8* and *TROAP* additionally showed higher expression in advanced-stage tumors (Stage III/IV vs. Stage I/II; *p* = 0.015 and *p* = 0.022, respectively; Figure S3, in Supplementary Materials). Taken together, these exploratory findings highlight *CDCA8* and *TROAP* as promising candidates for further evaluation as prognostic biomarkers of aggressive disease biology in ccRCC.

### 3.4. Prognostic Relevance of Candidate Markers in Metastatic ccRCC

**Figure 4:**
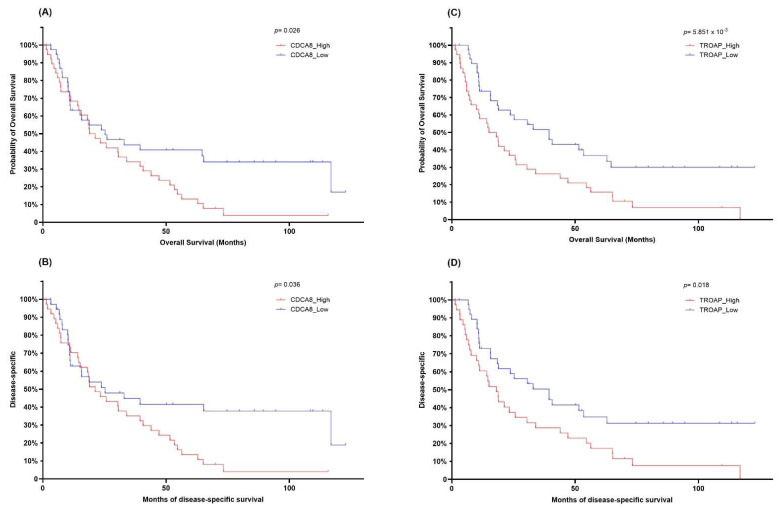
Prognostic relevance of *CDCA8* and *TROAP* in metastatic ccRCC patients. (**A**,**B**) Kaplan–Meier survival curves for overall survival (OS) and disease-specific survival (DSS) stratified by *CDCA8* expression levels in TCGA patients presenting with metastatic disease (M1). Patients in the high-expression group exhibited significantly worse OS (*p* = 0.026) and DSS (*p* = 0.036). (**C**,**D**) Kaplan–Meier curves for OS and DSS according to *TROAP* expression levels in the same cohort. High *TROAP* expression was associated with poor prognosis in both OS (*p* = 5.85 × 10^−3^) and DSS (*p* = 0.018).

Before validating prognostic associations in the TCGA cohort, we assessed whether bulk transcriptomic differences could be explained by tumor microenvironmental composition. Deconvolution using ssGSEA and ESTIMATE showed no systematic bias in stromal or immune fractions, apart from modest variation in fibroblast and endothelial scores (Figure S4, in Supplementary Materials). To further examine the prognostic implications of the ROC-selected candidates, survival analyses were performed in the TCGA ccRCC cohort restricted to patients presenting with metastatic disease (M1). This design provided a complementary validation setting compared with the discovery cohort, allowing assessment of whether candidate markers identified in initially metastasis-free tumors also carried relevance in advanced disease. Importantly, this analysis was intended as a prognostic evaluation rather than a prediction of postoperative recurrence. Among the five genes, only *CDCA8* and *TROAP* consistently demonstrated significant survival associations. In Kaplan–Meier analyses, patients with higher expression of *CDCA8* or *TROAP* showed significantly shorter OS and DSS compared with those with lower expression (Figure 4). These KM curves were used to illustrate that only *CDCA8* and *TROAP* retained prognostic signal among the five ROC-selected candidates. By contrast, *BASP1*, *KIF2C*, and *LMNB1* displayed strong discriminatory capacity in the discovery ROC analysis (AUC > 0.9) but failed to stratify OS or DSS outcomes consistently in the TCGA dataset (Figure S5, in Supplementary Materials). This inconsistency underscores the importance of external validation and suggests that only a subset of candidates maintain robust prognostic value in advanced disease.

### 3.5. TROAP and CDCA8 Remain Independent Prognostic Factors in Multivariate Analysis

**Figure 5:**
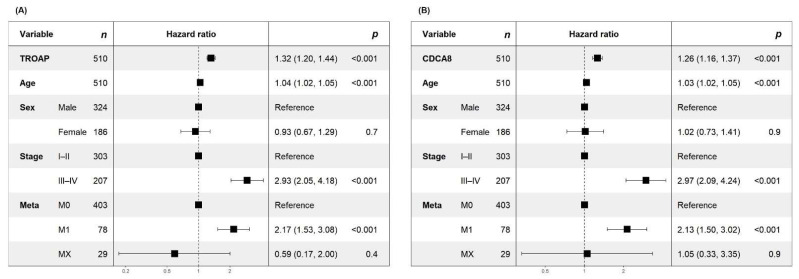
Multivariable continuous Cox regression in TCGA ccRCC. (**A**) Forest plot of the multivariable Cox proportional-hazards model for *TROAP*, showing hazard ratios (HRs) per SD increase in expression, adjusted for age, sex, AJCC stage (III–IV vs. I–II), and metastasis status (M1 vs. M0). (**B**) Corresponding model for *CDCA8* with the same covariates. Point estimates represent HRs, and horizontal bars indicate 95% confidence intervals.

While Kaplan–Meier analyses provided an initial illustration of survival differences, such dichotomized approaches are limited by arbitrary cut-offs and loss of information. To more rigorously evaluate prognostic effects, we therefore applied multivariable continuous Cox proportional-hazards models in the TCGA ccRCC cohort, adjusting for metastasis status (M1 vs. M0), age, sex, and AJCC stage. Modeling gene expression per SD increase enabled effect estimates that avoid threshold-dependent bias. In these fully adjusted models, both genes retained robust prognostic associations with overall survival (OS). Specifically, higher expression was associated with increased mortality for *TROAP* (HR per SD, 1.32, 95% CI 1.20–1.44, *p* < 0.001) and *CDCA8* (HR per SD, 1.26, 95% CI 1.16–1.37, *p* < 0.001), independent of stage and baseline metastatic status (Figure 5). The model C-index was 0.77, indicating good discriminative performance. Full OS and DSS results including N, number of events, and covariate effects—are provided in Table S3 (in Supplementary Materials). Because the objective of this analysis was to assess prognosis rather than to develop a tool for postoperative risk prediction, interpretation was focused on patients with advanced disease, where survival outcomes can be meaningfully evaluated. This framing underscores that *TROAP* and *CDCA8* are best understood as prognostic indicators of aggressive disease biology, rather than as markers intended for predicting latent recurrence.

## 4. Discussion

The risk of postoperative metastasis in ccRCC remains a major clinical challenge, as conventional staging and grading fail to fully capture the latent metastatic potential of ostensibly localized tumors [35,36]. In this study, transcriptomic alterations enriched for mitotic control and chromosomal stability pathways were associated with later metastatic progression, suggesting that aggressive biology may be detectable at the time of surgery. Although the discovery cohort included only four metastatic events, rigorous statistical thresholds, optimism-corrected ROC analyses, and external validation in TCGA lend support to the robustness of the observations. Among the nominated hub genes, *CDCA8* and *TROAP* consistently emerged as markers of aggressive disease biology, demonstrating associations with subsequent metastasis in initially non-metastatic patients and with adverse survival among patients presenting with metastatic disease. Their performance across these distinct clinical contexts suggests that they capture intrinsic metastatic competence rather than simply reflecting tumor burden. Several limitations must be acknowledged. The small number of metastatic events in the discovery set inevitably raises the risk of optimistic bias, even with multiple-testing control and bootstrap correction. Validation in TCGA was restricted to patients annotated as metastatic at baseline (M1), leading to a stage mismatch that shifts the question from predicting latent recurrence to assessing prognosis in established metastasis. This reflects an inherent limitation of the TCGA dataset, which does not provide uniformly curated information on the timing of metastasis. Furthermore, bulk RNA sequencing collapses tumor-intrinsic and microenvironmental contributions into aggregate signals. Although QC-annotated PCA and deconvolution analyses indicated that technical and compositional effects were limited, residual confounding cannot be excluded. For these reasons, the findings should be regarded as exploratory and hypothesis-generating rather than definitive. Nevertheless, the results support the prognostic potential of *CDCA8* and *TROAP*. Both genes remained significantly associated with adverse outcomes in continuous multivariable Cox models, adjusted for stage and metastasis status, indicating prognostic value beyond stage correlation. Mechanistically, *CDCA8* and *TROAP* converge on pathways of mitotic fidelity and chromosomal segregation, processes that may promote chromosomal instability, tumor evolution, and therapeutic resistance. Prior studies have linked these genes to poor outcomes in other malignancies, underscoring their cross-cancer relevance [37,38,39]. In ccRCC specifically, cell-cycle dysregulation may intersect with oncogenic signaling such as mTOR activation, a central driver of progression and therapeutic targeting [40,41,42]. Moreover, emerging evidence highlights the contribution of mitochondrial DNA mutations and altered mitochondrial dynamics to ccRCC progression, raising the possibility that mitotic dysregulation and mitochondrial stress responses interact to facilitate dissemination [43,44,45,46]. Taken together, these findings suggest that *CDCA8* and *TROAP* may serve as candidate prognostic biomarkers of metastatic competence in ccRCC. Larger prospective studies with curated recurrence endpoints, multi-institutional validation, and orthogonal approaches such as immunohistochemistry, single-cell, or spatial transcriptomics will be essential to confirm their clinical and biological significance.

## 5. Conclusions

This study identified transcriptomic signatures associated with postoperative metastatic progression in ccRCC, particularly involving mitotic and chromosomal stability pathways. Among the candidate hub genes, *CDCA8* and *TROAP* consistently emerged as markers of aggressive disease biology. Both genes were upregulated in patients who later developed metastasis in the discovery cohort and were independently associated with adverse survival in TCGA when analyzed as continuous variables adjusted for clinicopathologic factors. Although the analyses were exploratory, the results suggest that *CDCA8* and *TROAP* capture intrinsic metastatic competence and may provide prognostic information complementary to conventional staging. By linking molecular alterations detectable at the time of surgery to survival outcomes in advanced disease, this work highlights the potential of transcriptomic profiling to refine postoperative risk stratification. With validation in larger, prospective cohorts and further functional investigation, these biomarkers may contribute to improved patient management and a deeper understanding of ccRCC progression.

## Data Availability

RNA-seq datasets used for survival analyses are publicly available from The Cancer Genome Atlas (TCGA) via https://portal.gdc.cancer.gov (accessed on 15 May 2025). TMM-normalized gene expression values for the five biomarker candidates are provided in Supplementary Table S4. Additional experimental data are available from the corresponding author upon reasonable request. These data are not publicly available due to privacy and ethical restrictions.

## Supplementary Materials

The following supporting information can be downloaded at: https://www.mdpi.com/article/10.3390/cancers17182975/s1, Figure S1. Principal component analysis (PCA) plots annotated by sequencing quality control (QC) metrics (read count and GC%); Figure S2. Protein–protein interaction (PPI) network of differentially expressed genes (DEGs) and hub gene identification; Figure S3. Stage-wise distribution and subgroup bar plots of candidate gene expression; Figure S4. Immune and stromal deconvolution results based on ssGSEA and ESTIMATE scores; Figure S5. Kaplan–Meier survival curves for *BASP1*, *KIF2C*, and *LMNB1* in the discovery cohort; Table S1. Clinical characteristics of the discovery cohort (M0 vs. M1; *n* = 30); Table S2. Spearman correlation coefficients between the first five principal components (PC1–PC5) and sequencing QC metrics (total read count and GC%); Table S3. Continuous Cox regression analyses in TCGA ccRCC (per-SD hazard ratios, adjusted for age, sex, stage, and metastasis status); Table S4. Gene expression matrix of candidate genes (*BASP1*, *CDCA8*, *KIF2C*, *LMNB1*, *TROAP*) in the discovery cohort (M0 vs. M1).
