# Supplementary material for: CDCA8 and TROAP as Prognostic Biomarkers of Postoperative Metastatic Progression in Clear Cell Renal Cell Carcinoma
Source: Cancers (Basel). 2025 Sep 11;17(18):2975. doi: 10.3390/cancers17182975 (PMC12468399; doi:10.3390/cancers17182975)
Supplement: Supplementary file 1 [file cancers-17-02975-s001.zip › Figure S1.pdf]

Supplementary Figure S1. Principal component analysis (PCA) annotated with sequencing quality control metrics.

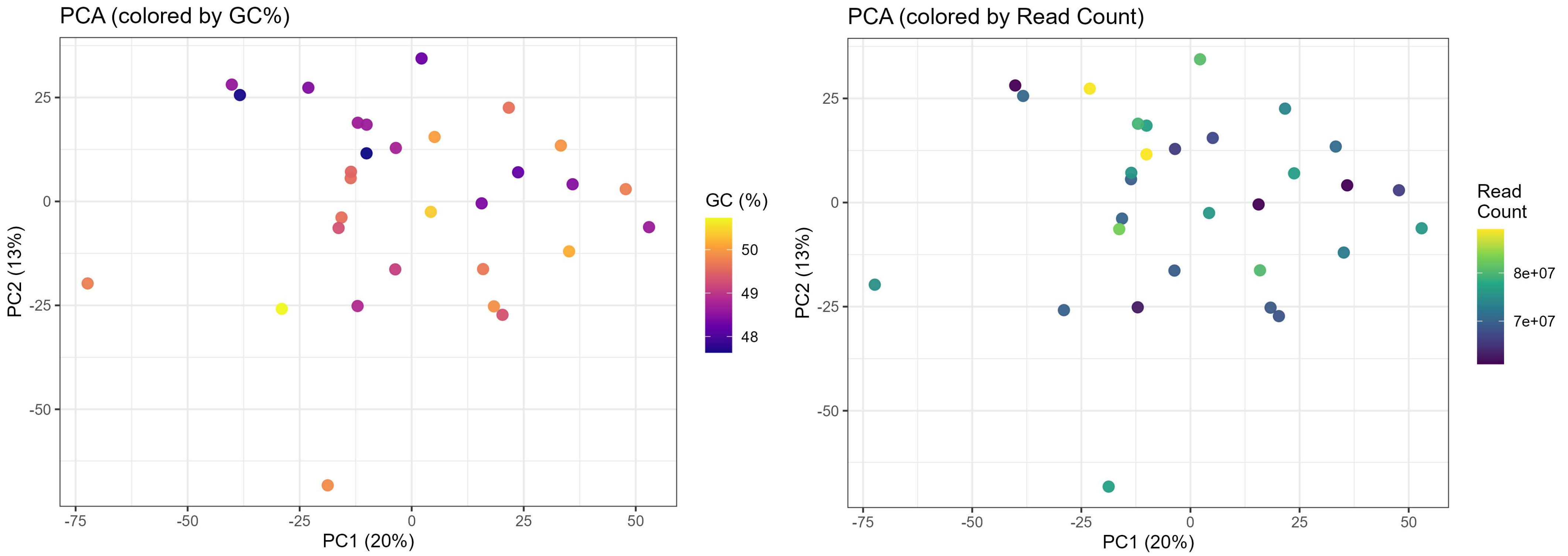

Figure S1. PCA based on TMM-normalized expression values, annotated with sequencing QC metrics. (A) Samples colored by total read count. (B) Samples colored by GC content. No clustering was observed according to these technical factors.
