# Supplementary material for: CDCA8 and TROAP as Prognostic Biomarkers of Postoperative Metastatic Progression in Clear Cell Renal Cell Carcinoma
Source: Cancers (Basel). 2025 Sep 11;17(18):2975. doi: 10.3390/cancers17182975 (PMC12468399; doi:10.3390/cancers17182975)
Supplement: Supplementary file 1 [file cancers-17-02975-s001.zip › Figure S2.pdf]

Supplementary Figure 2. Protein–Protein Interaction (PPI) Network of Metastasis-Associated Genes

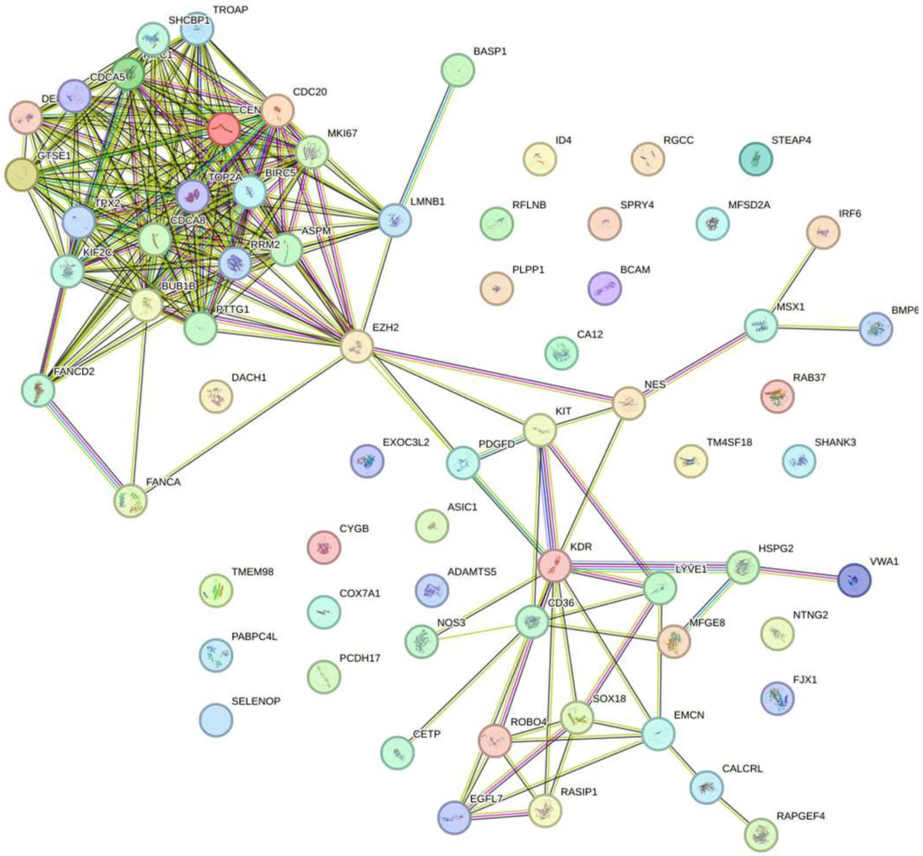

Figure S2. STRING-based protein–protein interaction (PPI) network constructed from 69 differentially expressed genes (DEGs) between M1 and M0 groups. Nodes represent proteins encoded by DEGs, and edges indicate predicted interactions with a confidence score > 0.4. A densely connected subnetwork enriched for mitotic and chromosomal regulation-related genes is visible in the upper left cluster, highlighting potential molecular drivers of metastatic progression.
