# Supplementary material for: CDCA8 and TROAP as Prognostic Biomarkers of Postoperative Metastatic Progression in Clear Cell Renal Cell Carcinoma
Source: Cancers (Basel). 2025 Sep 11;17(18):2975. doi: 10.3390/cancers17182975 (PMC12468399; doi:10.3390/cancers17182975)
Supplement: Supplementary file 1 [file cancers-17-02975-s001.zip › Figure S3.pdf]

Supplementary Figure 3. Stage-wise Expression of *CDCA8* and *TROAP* in Tumor Samples from the Institutional ccRCC Cohort

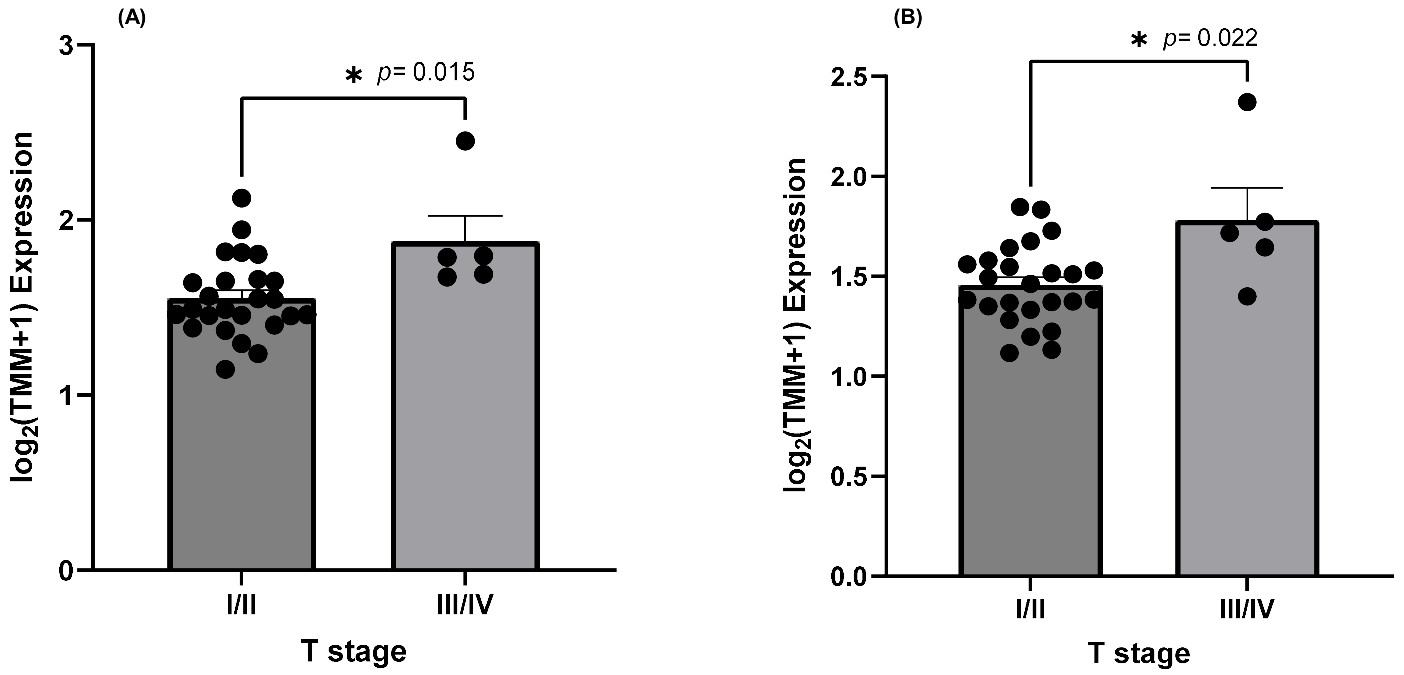

Figure S3. Expression of *CDCA8* and *TROAP* according to pathological T stage (I/II vs III/IV) in tumor samples from the institutional ccRCC cohort. (A) *CDCA8* and (B) *TROAP* expression according to pathological T stage (I/II vs III/IV). Expression values were TMM-normalized and log_2-transformed (log_2[TMM+1]). Bars represent mean ± SEM, and individual patients are shown as dots. Statistical comparisons were performed using the Mann–Whitney test; $p = 0.015$ for *CDCA8* and $p = 0.022$ for *TROAP*.
