# Supplementary material for: CDCA8 and TROAP as Prognostic Biomarkers of Postoperative Metastatic Progression in Clear Cell Renal Cell Carcinoma
Source: Cancers (Basel). 2025 Sep 11;17(18):2975. doi: 10.3390/cancers17182975 (PMC12468399; doi:10.3390/cancers17182975)
Supplement: Supplementary file 1 [file cancers-17-02975-s001.zip › Figure S4.pdf]

Supplementary Figure 4. Tumor microenvironment (TME) composition analysis by ssGSEA and ESTIMATE in the institutional ccRCC cohort.

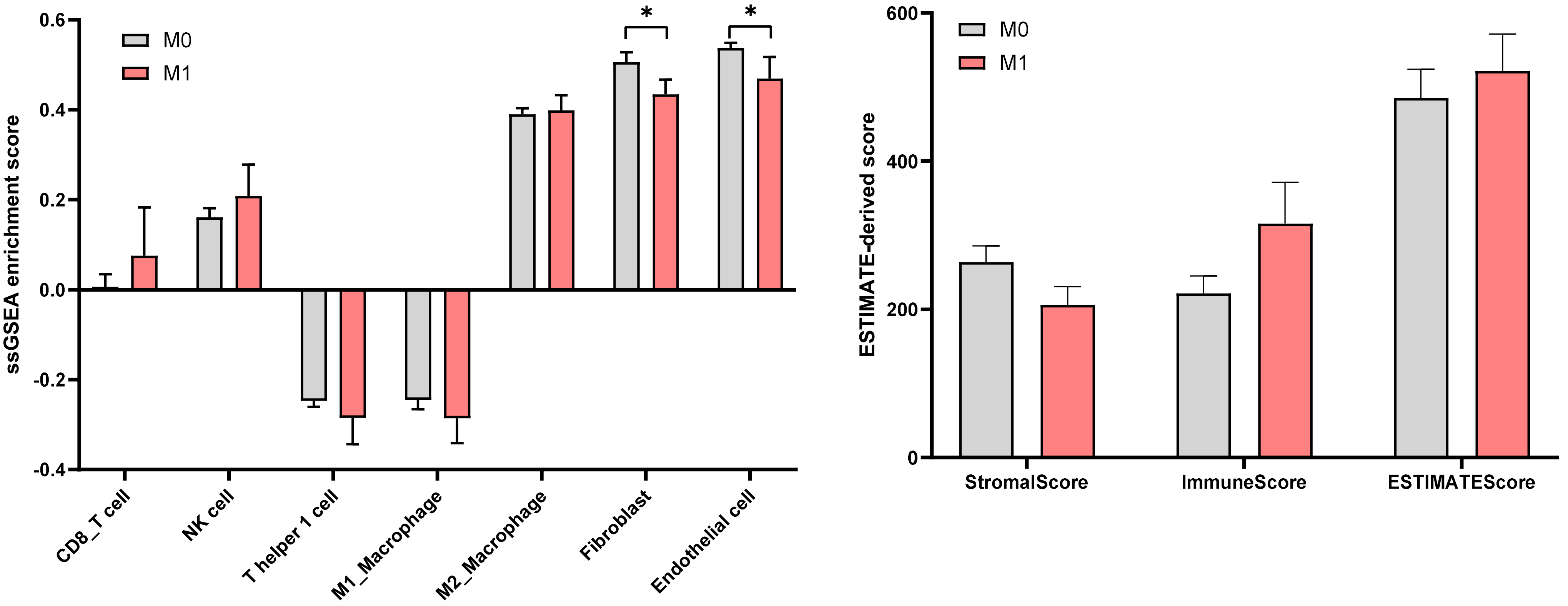

Figure S4. Tumor microenvironmental composition was assessed to evaluate whether transcriptomic differences could be attributed to variation in stromal or immune fractions. (A) Single-sample gene set enrichment analysis (ssGSEA) enrichment scores of selected immune and stromal cell subsets, comparing non-metastatic (M0) and metastatic (M1) tumors. Significant differences were observed in fibroblast and endothelial cell scores (*$p < 0.05$). (B) ESTIMATE-derived StromalScore, ImmuneScore, and ESTIMATEScore across M0 and M1 tumors. No significant differences were detected in overall stromal or immune content between groups.
