# Supplementary material for: CDCA8 and TROAP as Prognostic Biomarkers of Postoperative Metastatic Progression in Clear Cell Renal Cell Carcinoma
Source: Cancers (Basel). 2025 Sep 11;17(18):2975. doi: 10.3390/cancers17182975 (PMC12468399; doi:10.3390/cancers17182975)
Supplement: Supplementary file 1 [file cancers-17-02975-s001.zip › Figure S5.pdf]

Supplementary Figure 5. Survival Analysis of *BASP1*, *KIF2C*, and *LMNB1* in Metastatic ccRCC Patients

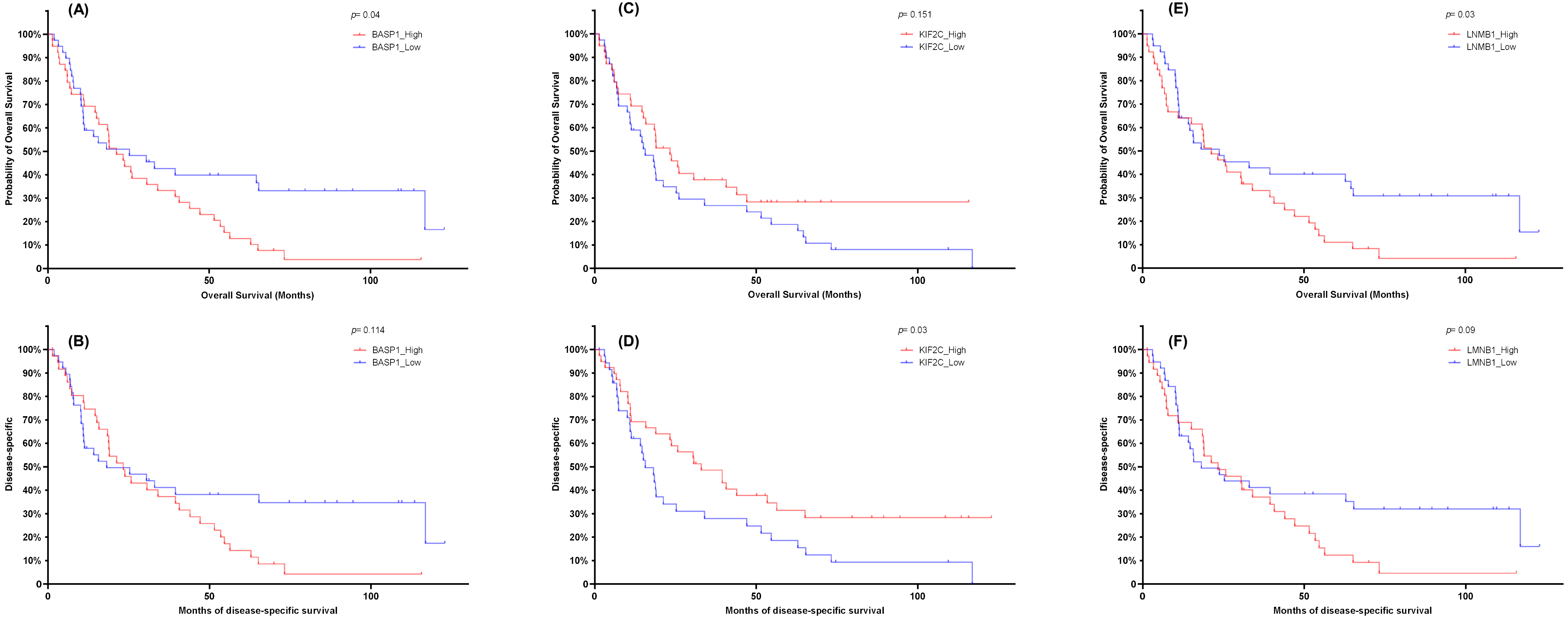

Figure S5. Kaplan–Meier curves for overall survival (OS) and disease-specific survival (DSS) stratified by the expression of *BASP1*, *KIF2C*, and *LMNB1* in patients with metastatic clear cell renal cell carcinoma (ccRCC) from the TCGA cohort. Patients were divided into high and low expression groups based on the median transcript level of each gene. Although each gene showed significant ROC performance in earlier analyses, only one of the two survival endpoints (OS or DSS) reached statistical significance in each case, thereby limiting their utility as robust prognostic markers. (A–B) *BASP1*, (C–D) *KIF2C*, and (E–F) *LMNB1*.
