# Supplementary material for: CDCA8 and TROAP as Prognostic Biomarkers of Postoperative Metastatic Progression in Clear Cell Renal Cell Carcinoma
Source: Cancers (Basel). 2025 Sep 11;17(18):2975. doi: 10.3390/cancers17182975 (PMC12468399; doi:10.3390/cancers17182975)
Supplement: Supplementary file 1 [file cancers-17-02975-s001.zip › Table S1.docx]

|  | **M1 group (n=4)** | **M0 group (n=26)** |
| --- | --- | --- |
| **Age** |  |  |
| Median | 74 | 63 |
| Range | 56 - 77 | 42 - 82 |
| **Gender** |  |  |
| Male | 4 (100 %) | 17 (65.4%) |
| Female | 0 (0%) | 9 (34.6%) |
| **Stage** |  |  |
| I | 1 (25%) | 24 (92.3%) |
| II | 1 (25%) | 3 (7.7%) |
| III | 2 (50%) | 0 (0%) |
| IV | 0 (0%) | 0 (0%) |
| **Grade** |  |  |
| I | 0 (0%) | 0 (0%) |
| II | 0 (0%) | 15 (57.7%) |
| III | 3 (75%) | 11 (42.3%) |
| IV | 1 (15%) | 0 (0%) |
| **Follow-up** |  |  |
| Diagnosis → metastasis | Pt1: 2022-03-31 → 2023-10-11  Pt2: 2022-03-31 → 2024-12-18  Pt3: 2022-06-09 → 2023-05-14  Pt4: 2022-08-04 → 2024-11-27 | Median 24 month (range 14–34),  no metastasis |

Table S1. Clinical Characteristics M1 group and M0 group
