# Supplementary material for: CDCA8 and TROAP as Prognostic Biomarkers of Postoperative Metastatic Progression in Clear Cell Renal Cell Carcinoma
Source: Cancers (Basel). 2025 Sep 11;17(18):2975. doi: 10.3390/cancers17182975 (PMC12468399; doi:10.3390/cancers17182975)
Supplement: Supplementary file 1 [file cancers-17-02975-s001.zip › Table S2.docx]

Table S2. Spearman correlation coefficients between PC1–PC5 and sequencing quality control metrics (total read count and GC%).

| **PC** | **Variable** | **Spearman_rho** | **p_value** | **n** | **q_FDR** |
| --- | --- | --- | --- | --- | --- |
| PC1 | read_count | -0.16707 | 0.375952 | 30 | 0.559665 |
| PC1 | gc | 0.096785 | 0.610903 | 30 | 0.678781 |
| PC2 | read_count | 0.156841 | 0.406223 | 30 | 0.559665 |
| PC2 | gc | -0.49527 | 0.00539 | 30 | 0.026948 |
| PC3 | read_count | 0.143493 | 0.447732 | 30 | 0.559665 |
| PC3 | gc | 0.168873 | 0.372348 | 30 | 0.559665 |
| PC4 | read_count | -0.51591 | 0.003964 | 30 | 0.026948 |
| PC4 | gc | -0.23162 | 0.218121 | 30 | 0.559665 |
| PC5 | read_count | 0.171969 | 0.36196 | 30 | 0.559665 |
| PC5 | gc | 0.060073 | 0.752504 | 30 | 0.752504 |
