# Supplementary material for: CDCA8 and TROAP as Prognostic Biomarkers of Postoperative Metastatic Progression in Clear Cell Renal Cell Carcinoma
Source: Cancers (Basel). 2025 Sep 11;17(18):2975. doi: 10.3390/cancers17182975 (PMC12468399; doi:10.3390/cancers17182975)
Supplement: Supplementary file 1 [file cancers-17-02975-s001.zip › Table S3.docx]

| **Endpoint** | **Gene** | **HR**  **(per SD)** | **95% CI** | **p-value** | **N** | **Events** | **Covariates** |
| --- | --- | --- | --- | --- | --- | --- | --- |
| **OS** | CDCA8 | 1.27 | 1.18–1.38 | <0.001 | 510 | 169 | age, sex, stage, metastasis |
| **OS** | TROAP | 1.33 | 1.22–1.46 | <0.001 | 510 | 169 | age, sex, stage, metastasis |
| **DSS** | CDCA8 | 1.31 | 1.20–1.43 | <0.001 | 500 | 108 | age, sex, stage, metastasis |
| **DSS** | TROAP | 1.39 | 1.26–1.53 | <0.001 | 500 | 108 | age, sex, stage, metastasis |

Table S3. Continuous Cox regression (per-SD) for TCGA ccRCC.
