# Supplementary material for: CDCA8 and TROAP as Prognostic Biomarkers of Postoperative Metastatic Progression in Clear Cell Renal Cell Carcinoma
Source: Cancers (Basel). 2025 Sep 11;17(18):2975. doi: 10.3390/cancers17182975 (PMC12468399; doi:10.3390/cancers17182975)
Supplement: Supplementary file 1 [file cancers-17-02975-s001.zip › Table S4.docx]

Table S4. Gene Expression Analysis

| M0 group | | | | | |
| --- | --- | --- | --- | --- | --- |
|  | BASP1 | CDCA8 | KIF2C | LMNB1 | TROAP |
| Sample1 | 1.750774 | 1.8592587 | 1.7016613 | 2.9452219 | 1.750774 |
| Sample2 | 3.6663222 | 1.5921042 | 1.8432722 | 3.3019137 | 1.8099591 |
| Sample3 | 3.1794955 | 1.586605 | 2.1890081 | 3.2466221 | 1.6086437 |
| Sample4 | 3.3311857 | 1.7560984 | 1.9012639 | 4.3902708 | 1.9218318 |
| Sample5 | 3.6717885 | 1.6098718 | 2.3997049 | 3.7844118 | 1.9301928 |
| Sample6 | 3.1367688 | 1.2929756 | 1.3600122 | 3.4106572 | 1.4516544 |
| Sample7 | 3.1421956 | 1.9879954 | 1.5988156 | 3.1792497 | 2.1398287 |
| Sample8 | 4.2946227 | 1.9487668 | 1.8364032 | 3.9492407 | 1.7425267 |
| Sample9 | 3.0597537 | 1.8879991 | 1.7912337 | 3.6775429 | 1.6396731 |
| Sample10 | 4.2558554 | 1.3347776 | 1.1234052 | 3.5869678 | 1.956442 |
| Sample11 | 4.1072794 | 1.6070072 | 2.0379543 | 3.633534 | 1.5856286 |
| Sample12 | 3.4601148 | 1.5496565 | 1.2793012 | 3.1396155 | 1.3559385 |
| Sample13 | 2.3464813 | 1.4302811 | 1.134494 | 2.842645 | 1.2143173 |
| Sample14 | 5.355856 | 2.5663871 | 3.2717418 | 4.7565396 | 2.8491839 |
| Sample15 | 3.0710731 | 2.2842066 | 2.447494 | 4.3548988 | 2.4716671 |
| Sample16 | 4.4190621 | 2.1194464 | 2.1068415 | 3.0123077 | 1.7413937 |
| Sample17 | 5.4981719 | 1.6387454 | 2.1116724 | 4.2742989 | 2.1923899 |
| Sample18 | 3.2634703 | 1.8505688 | 1.8975005 | 3.6535307 | 2.1398262 |
| Sample19 | 2.8613496 | 2.3131908 | 2.6469332 | 3.4287333 | 2.5180539 |
| Sample20 | 4.928122 | 1.9223944 | 2.242913 | 4.2149509 | 2.1205575 |
| Sample21 | 3.9775744 | 1.8151006 | 2.2619081 | 3.9152839 | 2.1610502 |
| Sample22 | 3.0678232 | 1.1923883 | 0.8353222 | 3.1265814 | 1.7546957 |
| Sample23 | 4.1741803 | 1.5187906 | 1.4454971 | 3.3881175 | 1.7363382 |
| Sample24 | 3.9661803 | 1.5833499 | 1.7572876 | 3.7235884 | 1.805627 |
| Sample25 | 4.4678891 | 1.1697252 | 1.7991499 | 3.6958332 | 2.4490795 |
| Sample26 | 4.6899024 | 2.4143218 | 2.7615751 | 4.3271193 | 2.5254067 |
|  |  |  |  |  |  |
| M1 group | | | | | |
|  | BASP1 | CDCA8 | KIF2C | LMNB1 | TROAP |
| Sample1 | 4.6406371 | 2.1908179 | 2.8159408 | 4.4314042 | 2.4932331 |
| Sample2 | 4.6152103 | 2.1274853 | 2.3544486 | 4.5989746 | 2.2274325 |
| Sample3 | 6.7499824 | 4.1756993 | 4.7525716 | 6.1201806 | 4.4696484 |
| Sample4 | 6.2370553 | 2.5961645 | 2.8509227 | 4.161249 | 3.3617986 |
