# Supplementary figures and images for: CDCA8 and TROAP as Prognostic Biomarkers of Postoperative Metastatic Progression in Clear Cell Renal Cell Carcinoma
Source: Cancers (Basel). 2025 Sep 11;17(18):2975. doi: 10.3390/cancers17182975 (PMC12468399; doi:10.3390/cancers17182975)

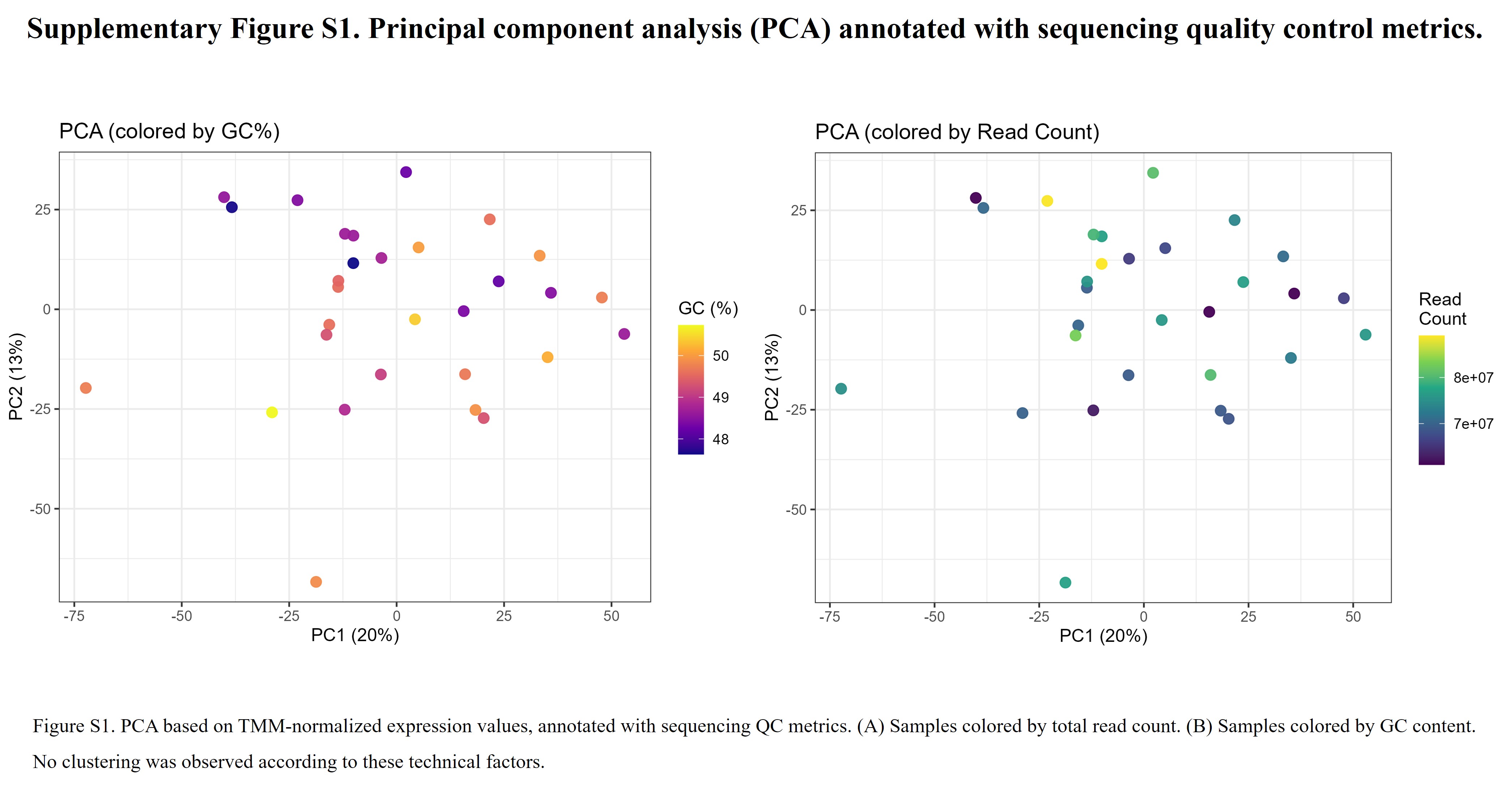

Supplement: Supplementary file 1 [file cancers-17-02975-s001.zip › Figure S1.jpg]

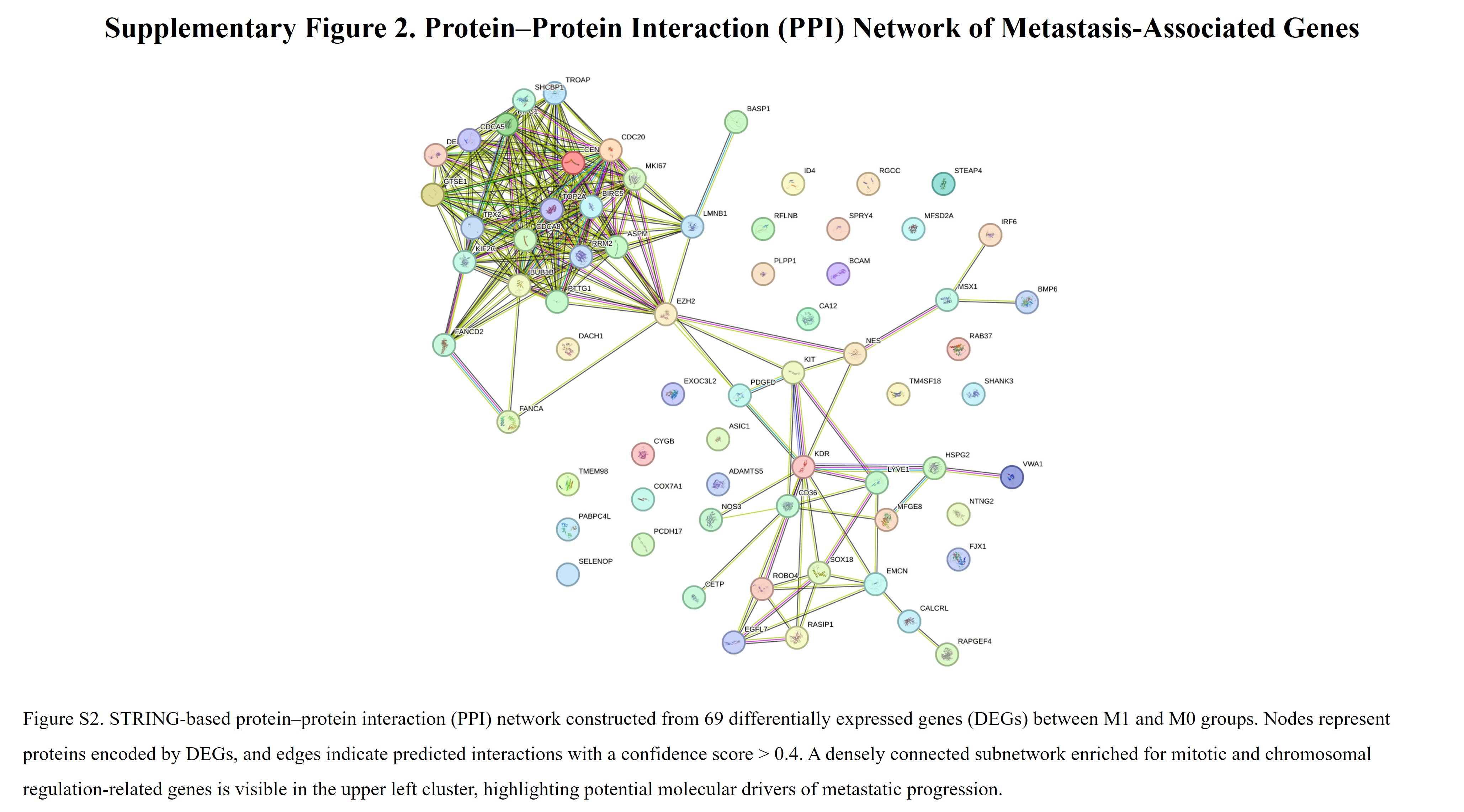

Supplement: Supplementary file 1 [file cancers-17-02975-s001.zip › Figure S2.jpg]

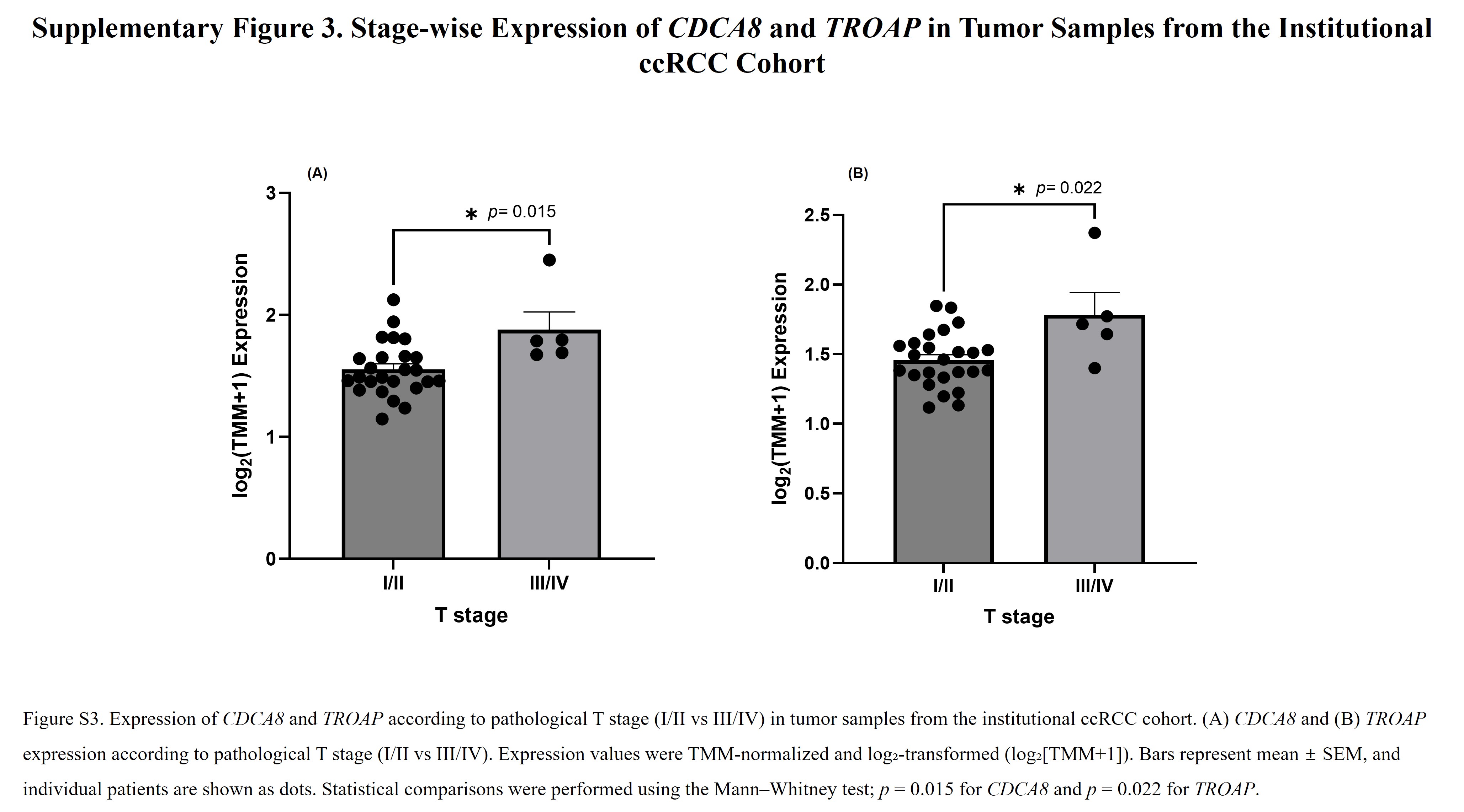

Supplement: Supplementary file 1 [file cancers-17-02975-s001.zip › Figure S3.jpg]

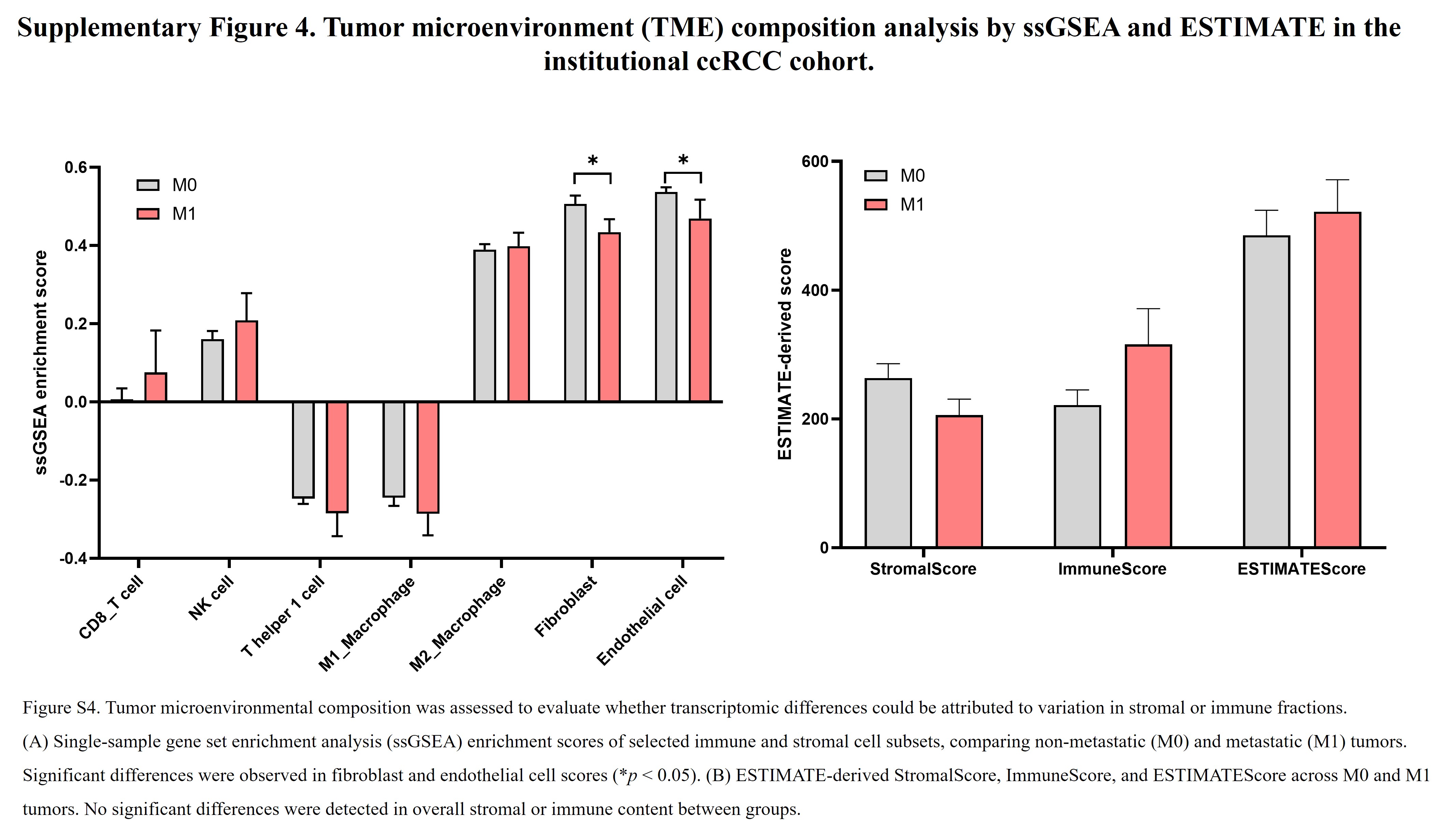

Supplement: Supplementary file 1 [file cancers-17-02975-s001.zip › Figure S4.jpg]

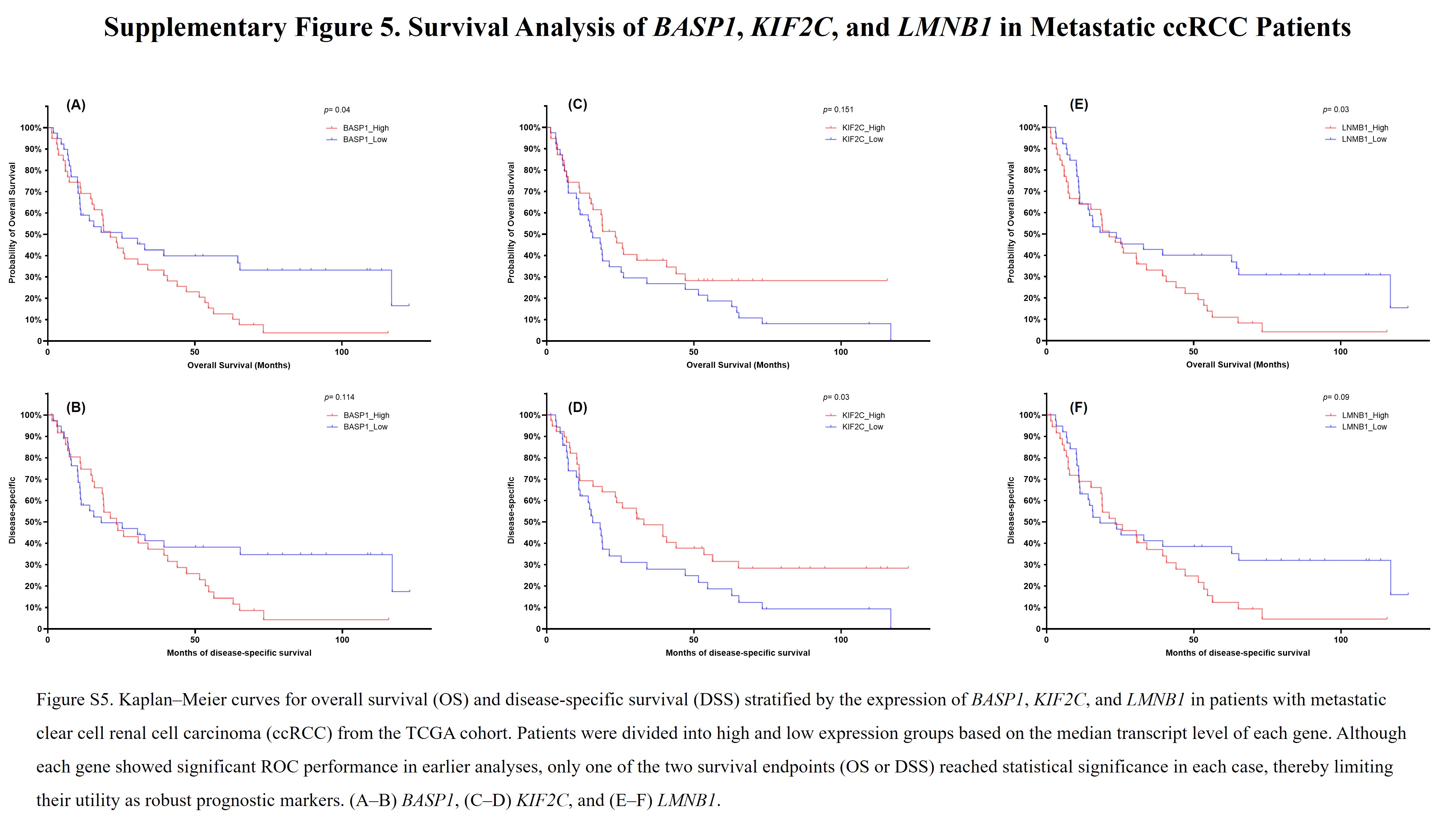

Supplement: Supplementary file 1 [file cancers-17-02975-s001.zip › Figure S5.jpg]
